# Prevalence of Vitamin D Deficiency in Patients Presenting with an Orthopaedic Trauma at a Tertiary Centre in South India - Implications and Protocols for Replacement Therapy

**DOI:** 10.5704/MOJ.1507.007

**Published:** 2015-07

**Authors:** VM Cherian, M Gouse, s Albert, V Jayasankar

**Affiliations:** Department of Orthopaedics, Christian Medical College, Vellore, India

## Introduction

Vitamin D has been the focus of much scientific literature in recent years owing to various studies showing its association with a wide variety of pathological conditions^1,2^. Sun exposure, diet and fortified supplementation account for a bulk of Vitamin D intake in humans. Activation of vitamin D is sequential and requires sun exposure for conversion of 7-dehydrocholesterol to Vitamin D3. Further metabolism in the liver converts Vitamin D3 to 25 –hydroxyvitamin D3. Conversion to its active form 1,25 dihydroxyvitamin D3 (Calcitriol) occurs in the kidneys^2^. The importance of Vitamin D in calcium metabolism and bone health is well known and documented. Controversies exist regarding the true prevalence of hypovitaminosis, however in developing countries the prevalence of vitamin D deficiency in all age groups is probably higher^3^. Adding to this the burden and morbidity of skeletal trauma, persistent deficiency may have a deleterious effect in the injured^4, 5^. Vitamin D, with its positive effect on bone health does play a role in the biology of fracture repair and remodelling^6^. The role of Vitamin D replacement as sole biological effectors in fracture repair may be difficult to quantify and confounded by other variables at play in bone healing. Studies looking at vitamin D levels in orthopaedic patients have also shown significant levels of deficiency and have put forward recommendations for evaluation and supplementation^7,8^.

Many studies in the last decade have associated Vitamin D with various diseases with varied results on their outcome^1,9,5^. It has been implicated in cancer biology, diabetes, hypertension, multiple sclerosis, slipped capital femoral epiphysis and even falls in the elderly to name a few^10,11,12,13^.

In western countries, fortified food products are a major source of Vitamin D^9^. Policies on food fortification with Vitamin D are yet to be established in many developing countries and the true prevalence of deficiency is not known. Few studies in select population groups and cohorts seem to reflect that prevalence in India is much higher than what would be expected^14,13^. We sought to determine the prevalence of Hypo-vitaminosis D in patients who presented with an orthopaedic injury and suggest a cost effective protocol if required for supplementation and follow up.

## Materials and Methods

This study was approved by the Instuitional Review Board (IRB Min .No 8393 dated 31.07.2013). We checked the Vitamin D levels of all patients who presented to the Accident and Emergency/ Orthopaedic Department Unit-1 with fractures from August 2013 to December 2013 Vellore. Christian Medical College (CMC) Vellore is a tertiary trauma care centre located in South India. We included patients in the age group 25 to 70 years who presented immediately following an orthopaedic injury the accident and emergency department. We excluded patients who presented after treatment at another centre, patients who were on prior supplementation of vitamin D and patients who presented with sepsis/ septic shock. We chose to exclude elderly patients as many would have been on supplements and their exposure to sunlight would have been considerably reduced. Patients who met our inclusion criteria, had along with other appropriate investigations, 25 hydroxyvitamin D3 levels were checked on collected samples using a Electro chemiluminescence immune assay system (Roche E- 170 modular system) in our in hospital clinical Biochemistry laboratory. Samples were obtained within a few hours of the injury prior to any administration of anaesthesia or any surgical procedure. Reviewing existing literature and recommendations we chose to classify our patients as follows^7^ severely deficient < 10 ng/mL, Moderately deficient 11-20ng/mL, Insufficient 21-32ng/mL, Adequate/ Normal > 32ng/mL. Those patients who are deficient were supplemented with vitamin D granules once a week for six to eight weeks.

## Statistical Analysis

The required sample size to find the prevalence of low Vitamin D among trauma patients was found to be 160 subjects with 7.5% precision, 95% confidence limits.

Descriptive statistics such as frequency and percentages were calculated for categorical variables. Summary measure such as mean /median with standard deviation / interquartile range was calculated for continuous variables. Vitamin d levels were associated with demographic variables using chi square test. P value less than 0.05 was considered to be statistics significant. Patient demographic data and the nature of orthopaedic injury sustained were also documented. Data was collected in a clinical research form, entered in Epidata V 3.1 and statistical analysis was performed using SPSS software version 17 (SPSS, Inc., Chicago, Illinois).

## Results

The average age of the patients in our population was 42.51(25-70 years). Patient demographic details are presented in Table I. All patients were from the same geographical region and presented within a few hours from the injury. The majority of the patients belong to a productive earning segment of the community and none of the patients were on prior calcium or Vitamin supplements.

**Table I:** Percentage of Vitamin D deficiency among male and female patients

SEX	Number	Percentage	Less than 10ng/dl	10-20ng/dl	21-32ng/dl	More than 32ng/dl	P- value
Male	122	76.3%	46(37.7%)	50(41%)	19(15.6%)	7(5.7%)	0.378
Female	38	23.7	19(50%)	15(39.5%)	3(7.9%)	1(2.6%)	

**Table II:** Prevalence of Vitamin D among patients with closed and open fractures

Injury-open/closed fractures	Number	percentage	Less than 10ng/dl.	10-20ng/dl	21-32ng/dl	More than 32ng/dl	P value
Open	50	31.3	18(36%)	19(38%)	10(20%)	3(6%)	0.458
Closed	110	68.8	47(42.7%)	46(41.8%)	12(10.9%)	5(4.5%)	

More male patients presented following injury, a representative sample of our injured population. Moreover being a tertiary centre, a significant number of our patients had open fractures.

Overall only 8 of 160 patients (5%) had adequate vitamin D levels (>32ng/dl). Breakdown of injury versus Vitamin D levels are given in Table III. It can be observed that prevalence of hypo-vitaminosis is uniformly distributed with the overall prevalence being 82% with the cut-off set at 20ng/dl and increasing to 95% when shifted to 32ng/dl. Fig 1.

**Figure:**
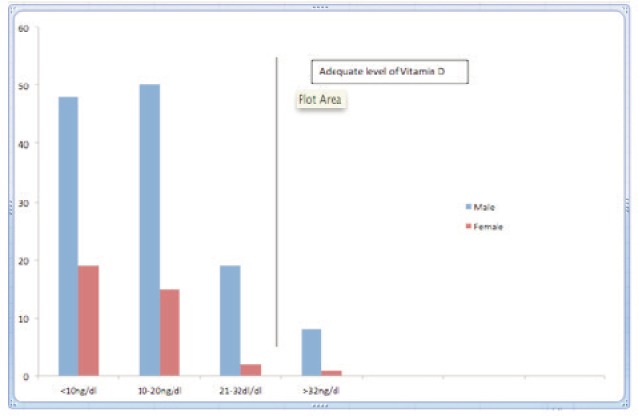


**Table III:** Vitamin D levels among patients with various injury patterns

Injury patterns	Number(percentage)	Less than 10ng/dl.	10-20ng/dl	21-32ng/dl	More than 32ng/dl
Pelvis/Acetab	4(2.5%)	1	2	1	0
Hip	26(16.3%)	10	12	4	0
Femur	14(8.8%)	3	6	3	2
Knee	8(5%)	2	5	1	0
Patella	1(0.6%)	1	0	0	0
Ankle	2(1.2%)	1	0	1	0
Tibia	16(10%)	4	7	3	2
Foot	18(11.3%)	5	8	3	2
Shoulder	3(1.9%)	1	0	2	0
Clavicle	3(1.9%)	1	2	0	0
Humerus	14(8.8%)	9	5	0	0
Elbow	1(0.6%)	0	0	0	1
Forearm	11(6.9%)	5	5	1	0
Wrist	13(8.1%)	6	5	1	0
Multiple injuries	26(16.3%)	16	8	2	0
Total	160	65	65	22	8

## Discussion

In south Indian weather is predominantly warm through the year and the population being universally dark skinned. The seasons through the year on the South Indian plains are once famously described as being “hot, hotter and hottest” through the year. Individual sun exposure is unlikely to be reduced during any part of the year. Despite controversies existing regarding the normal acceptable vitamin D level, various studies in otherwise asymptomatic cohorts world over have shown alarmingly low levels of Vitamin D^1,3,15,16^. What constitutes hypovitaminosis is also confusing with different studies using different cut off values to define deficiency. The cause of deficiency may be multi factorial ranging from absence fortification to ineffective sun exposure. The prevalence is bound to increase when the cut off for deficiency is lowered. What constitutes a normal level in a south Indian population has not been established. Most studies use levels recommended by either the Institute of Medicine (>20ng/dl) or the International osteoporosis foundation (>30ng/dl)^17,18^.

In our series the majority of the trauma patient was deficient of vitamin D (82%). We compared our result with other studies done in south India in various study population. The percentage of vitamin D deficiency among all studies was identical^19–22^.

The patients in this group may be considered to represent a productive cross section of the community considering the average age. None of the patients had been tested or treated for Vitamin D deficiency prior to their admission at our institution. Vitamin D deficiency in the adult patient need not always be associated with frank radiological signs of osteomalacia. Other signs and symptoms like vague bone pains and proximal muscle weakness are not always evaluated or treated and hence patients may be labelled as clinically normal^23,24^. Even if patients in this group were clinically asymptomatic, they may all benefit from supplementation post injury considering the benefit of Vitamin D on bone health, calcium metabolism and improved rehabilitative potential^24,25^. In our series all patient who had vitamin D deficient were supplements with calcirol granules sachet as recommended .M Brinker et at evaluated patients with an unexplained non-union and found that a majority of patients(84%) had a metabolic bone disorder, the commonest being Vitamin D deficiency and union occurred in some patients with just medical treatment^26^.

However the precise benefit of Vitamin D supplementation in every patient who have had skeletal injury in terms of infection control, prevention of non union and improved rehabilitation are difficult to assess^6,26,27^. However supplementation in this group would be beneficial in the absence of standardised food fortification programs. Dark skinned individuals are also at a slight disadvantage as the metabolism of vitamin D to its active form is reduced^2^.

The effect of Vitamin D on muscle power and function in a dose/level dependent manner is well known and replacement therapy in rehabilitative phase post trauma or surgery might be especially beneficial^2,28^. In patients planned to undergo Total hip replacement Functional scores were significantly lower in patients with low Vitamin D levels and showed improvement with replacement therapy^29^.

Hypervitaminosis with replacement therapy is a rare event and often described in literature as a result of accidental ingestion or a dispensing error. The upper safe limit of Vitamin D has not been established and upto 10,000 units per day may be safe^2,30^. The IOM (Institute of Medicine) has set 4,000units as a recommended daily intake level. Treatment protocols using 60,000 units once a week for six to eight weeks is unlikely to induce hypervitaminosis in patients who have received no prior therapy^17^.

## Conclusion

In this series the overall prevalence of Vitamin D deficiency is 95% which is abnormally high. Routine evaluation of Vitamin D in trauma patients may not be required in these trauma patients. Regular supplement of vitamin D therapy provided a favourable outcome in the trauma patients. Other modalities of Vitamin D supplementation such as, sun exposure and diet modification with fortification in this patient group in the recovery/ rehabilitative period post injury is of considerable importance.
